# Pain in Huntington’s disease and its potential mechanisms

**DOI:** 10.3389/fnagi.2023.1190563

**Published:** 2023-07-06

**Authors:** Jiajie Li, Yan Wang, Riyun Yang, Wenjun Ma, JunGuo Yan, Yi Li, Gang Chen, Jingying Pan

**Affiliations:** ^1^Department of Histology and Embryology, Medical School of Nantong University, Nantong, China; ^2^Center for Basic Medical Research, Medical School of Nantong University, Co-innovation Center of Neuroregeneration, Nantong, Jiangsu, China; ^3^Department of Anesthesiology, Affiliated Hospital of Nantong University, Nantong, Jiangsu, China

**Keywords:** Huntington disease, pain, prevalence, neuropathology, pain symptoms

## Abstract

Pain is common and frequent in many neurodegenerative diseases, although it has not received much attention. In Huntington’s disease (HD), pain is often ignored and under-researched because attention is more focused on motor and cognitive decline than psychiatric symptoms. In HD progression, pain symptoms are complex and involved in multiple etiologies, particularly mental issues such as apathy, anxiety and irritability. Because of psychiatric issues, HD patients rarely complain of pain, although their bodies show severe pain symptoms, ultimately resulting in insufficient awareness and lack of research. In HD, few studies have focused on pain and pain-related features. A detailed and systemic pain history is crucial to assess and explore pain pathophysiology in HD. This review provides an overview concentrating on pain-related factors in HD, including neuropathology, frequency, features, affecting factors and mechanisms. More attention and studies are still needed in this interesting field in the future.

## Introduction

Neurodegenerative diseases are critical problems that seriously affect the social economy and patients’ quality of life. Huntington’s disease (HD) is a genetic neurodegenerative disease represented by progressive dysfunction, including motor damage and psychiatric impairments (Dale and van Duijn, 2015; Maiuri et al., 2019), and has a 50% risk of being inherited by children (McColgan and Tabrizi, 2018). The average lifespan of HD is approximately 20 years from initial symptoms to death. To date, the main studies and treatments of HD focus on motor and cognitive problems, thereby ignoring pain issues, even though some patients complain about painful indications.

Pain is an unpleasant feeling or emotional injury, often identified through oral description, questionnaires or pain evaluation (de Tommaso et al., 2016a; Buhmann et al., 2020). Pain is a troublesome problem and has been extensively studied in Alzheimer’s disease (AD), Parkinson’s disease (PD) and motor neuron diseases (MND), with prevalence of 38–75%, 68–95% and 19–85%, respectively (de Tommaso et al., 2016a). Surprisingly, few articles about pain in HD have been reported, as pain is not considered a major theme because HD patients prefer to consult about motor issues rather than sensory problems. It is well-known that pain occurs in patients with normal sensory systems once they receive direct or indirect damage (Yam et al., 2018; Tsuda, 2021). Because of neurodegeneration, HD patients show sensory dysfunctions and abnormal pain behaviors that are rarely mentioned (de Tommaso et al., 2016a). In addition, chorea and cognitive symptoms deteriorate significantly as HD progresses, and patients with mental problems, such as issues with memory, emotion, and social cognition, find it difficult to describe their painful suffering, resulting in pain issues being greatly ignored and overlooked (Snowden, 2017; Achterberg et al., 2021).

This review intends to discuss research on pain in HD, including neuropathology, prevalence, symptoms, impacting factors and possible mechanisms. Overall, more studies and explorations of pain in HD are urgently required in the future.

## Epidemiology

It is difficult to obtain an exact prevalence estimation because HD is a rare disease with a great diversity of influencing factors. Because of geographical differences, epidemiological investigations often show under- or over-assessment of the total population (Snowden, 2017). Present data indicate that the average HD prevalence is approximately 5–10/100,000 in the UK but much higher in Scotland and England (Evans et al., 2013). The highest reported in North America is 13.7/100,000 (Fisher and Hayden, 2014; McColgan and Tabrizi, 2018). HD prevalence is higher in Europe than in Asia, at approximately 0.1–0.7/100,000 (Pringsheim et al., 2012; Sipila et al., 2015; Xu and Wu, 2015). In addition, lower prevalence is observed in black species rather than white and mixed species (McColgan and Tabrizi, 2018). The prevalence difference between ethnic groups might be closely associated with different HTT genotypes (Xu and Wu, 2015; Baig et al., 2016).

## Neuropathology

**FIGURE 1:**
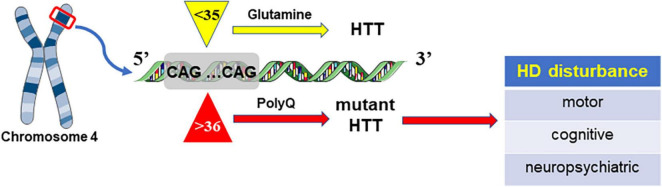
HD is caused by mutant Htt due to the increase in CAG repeats, which can lead to abnormal extension of polyglutamine (polyQ).

Huntingtin protein (Htt, 350kD) is abundantly expressed in the central nervous system, and its mutation can trigger HD. In general, there are approximately 17–20 CAG repeats in the Htt gene on chromosome 4 (Wyant et al., 2017). Abnormal CAG length is considered to be responsible for genic mutation and HD occurrence. Due to CAG expansion, the causative gene is considered to have neuronal toxicity and cause HD disease (McColgan and Tabrizi, 2018). There is a great correlation between CGA length, morbidity and progression: longer CGA repeats are usually linked with earlier onset and a higher rate. HD cases were reported to have more than 35 repeats, and people with a CAG size of 30–35 are deemed to gain little HD (Rubinsztein et al., 1996). Nevertheless, mutant gene carriers with more than 39 CAG repeats are predicted to develop disease and exhibit symptoms within several years (Rubinsztein et al., 1996; McColgan and Tabrizi, 2018; Figure 1).

HD is a brain-derived degenerative disease with widespread and progressive brain shrinkage, structural decline and functional impairment, especially in the striatum (Reiner et al., 1988; Fjodorova et al., 2015; Reiner and Deng, 2018). Although the striatum is the initial pathological site, there is still progressive damage in different cortical layers of the HD brain, from posterior to anterior, that generates symptoms during the whole lifespan (Rosas et al., 2002; Nana et al., 2014; Jimenez-Sanchez et al., 2017). Other areas, including the hippocampus, thalamus, and cerebellum, also undergo various levels of neuronal loss on the basis of HD stages. In addition, HD patients also display other symptoms, such as weight loss, muscle defects, and cardiac damage (Arenas et al., 1998; Gilbert, 2009). In general, males and females are impacted equally, and the average age of HD onset with symptoms is approximately 20–65 years, lasting about 20 years and then developing severe intellectual disability and eventually leading to death (Snowden, 2017; Wyant et al., 2017).

CAG length can expand when passed down from parents to children, especially when delivered through males, therefore leading to a high probability of HD symptoms appearing in childhood and adolescence prior to age 21, which is identified as Juvenile Huntington’s disease (JHD)(Reyes Molon et al., 2010; Quigley, 2017). JHD develops and progresses more rapidly than adult HD because of the extensive CAG repeats with a high mutation rate and longer length compared with their parents (Quarrell et al., 2012). Motor impairments remain the main symptoms in JHD, including chorea, gait changes, speech deficits and so on (Robertson et al., 2012; Snowden, 2017). Cognitive decline and psychiatric factors are also manifested in JHD, such as learning and memory problems, anxiety, aggression and depression, speech and language impairments (Fusilli et al., 2018; Cronin et al., 2019; Lesinskiene et al., 2020). Due to a series of functional deficiencies, suicide frequently occurs in JHD.

## Pain-related neuropathology

Mutant huntingtin (mHtt) can cause severe neuronal dysfunction, especially in medium spiny neurons (MSNs) in the striatum area, which is selectively susceptible to mHtt (Bohanna et al., 2008; Sprenger et al., 2019). Both autopsy reports and magnetic resonance imaging (MRI) have revealed tremendous atrophy in the striatum and cortical white matter (Bohanna et al., 2008; Tabrizi et al., 2011; McColgan and Tabrizi, 2018). Striatal degeneration can appear in the early HD phase, sometimes 10–15 years earlier than clinical detection (Paulsen et al., 2008; Wolf et al., 2013). It is well known that the striatum is a crucial part of the “pain matrix” and deals with various pain-related processes, such as sensorial discrimination, emotional processing, and cognitive evaluation (Fenton et al., 2015; Frediani and Bussone, 2019; Faraj et al., 2021). The striatum is principally responsible for affective- or cognitive- pain dimensions (Thompson and Neugebauer, 2019; Zhou et al., 2019; Tan and Kuner, 2021), which are crucial to determining the level of suffering from uncomfortable pain and then remembering, integrating and responding appropriately (Price, 2000; Nees and Becker, 2018). In addition, unusual sensory activation of the cortex has been discovered in HD (Boecker et al., 1999). Other brain areas associated with the “pain matrix” that are responsible for multiple pain dimensions include cortex, insula, thalamus, and somatosensory cortices (Apkarian et al., 2005; Perrotta et al., 2012; Cardenas Fernandez, 2015). Relevant to previous studies, different levels of atrophy have been discovered in the “pain matrix” regions of HD patient brains. Disease progression is greatly linked with the atrophy level in the HD brain (Bohanna et al., 2008; Coppen et al., 2016, 2018; Wilson et al., 2018; Johnson and Gregory, 2019). The basal ganglia are closely related to acute and chronic pain and show some changes in pain processing. In the mid stage of HD, an obvious delay in pain progression was found at the spinal cord level compared with the general population (De Tommaso et al., 2011; Perrotta et al., 2012; de Tommaso et al., 2016b). Overall, dysfunction at any degree and in any area has the possibility of causing unmodulated, transient or sustained pain.

## Pain-related prevalence

Pain has been studied and reported in AD, PD and other neurological disorders. However, pain in HD has rarely received attention, with only a few studies concerning pain issues. Several clinical studies have shown that pain in HD patients is greatly ignored and treated insufficiently, hence it is becoming a seriously undervalued issue (Andrich et al., 2009; de Tommaso et al., 2016a).

Pain development is closely influenced by psychological features, including depression, anxiety, apathy and irritability. Psychiatric symptoms occur frequently during all HD periods, especially the late stage, with an onset of approximately 33–76% (van Duijn et al., 2007, 2014). Some early studies reported that HD patients with depression had obviously painful symptoms; the greater the levels of anxiety and depression, the greater the severity of pain (Albin and Young, 1988; Arran et al., 2014). One study with 1474 HD gene carriers found that the pain prevalence ranged from 32% in the early period to 50% in the late period (Underwood et al., 2017), which was similar to another small sample study with a pain prevalence ranging from 11–62% (De Tommaso et al., 2011; Calvert et al., 2013). Another meta-analysis reported that the average pain prevalence in HD was about 41%, ranging from 36 to 46% (Sprenger et al., 2019). Recently, a worldwide pain-HD investigation showed that in HD mutation carriers, 34% had pain intervention, 17% underwent painful conditions, and 13% were treated with analgesics. For HD carriers without mutation, 42% had pain intervention and pain frequency was 12% in the middle period and 15% in the late period (Sprenger et al., 2021). The average prevalence of JHD in total HD cases is approximately 5–10% (Reyes Molon et al., 2010; Quigley, 2017; Lesinskiene et al., 2020).

## Pain-related symptoms

As an essential non-motor feature, pain has been undervalued and not linked with HD for a long time until a brief report in 1988 said that two HD cases had strong sensory symptoms. One patient described an unusual sensory feeling as intermittent “bee-sting pain”, which occasionally occurred on any body part, lasting several seconds or minutes. The pain frequency and intensity became worse in the following months, and rare therapeutic approaches could reduce his pain (Albin and Young, 1988). Another HD victim also suffered an uncomfortable sensation and intermittent, sharp-tingling pain in her arms and legs. She continued to complain of severe “lancinating pain” and auditory abnormality. Both eventually committed suicide (Albin and Young, 1988). These two cases only represent a small part of the HD population, indicating the essential role of sensory problems in HD, which cannot be overlooked. Some other studies have also shown abnormal sensory-induced potentials in HD patients (Oepen et al., 1981; Ehle et al., 1984). A marathon runner experienced serious muscle pain after running, which continued for several weeks and worsened his performance (Kosinski et al., 2007). In addition, a study with 90 sample, aimed to study pain in premanifest period HD and found that approximately 49% of them used narcotics, but only 14% used narcotics in the general population (Sprenger et al., 2019). The high use of narcotics indicates that pain is indeed a severe but unrecognized symptom in HD.

Studies concentrating on pain in HD display controversial results. In a study of HD with 19 cases, 11 patients estimated a high pain score, but only 3 of them obtained analgesia treatment (McColgan and Tabrizi, 2018). Another study with 28 HD samples reported that HD patients display a slowing pain development, and only 3 mild-stage patients expressed pain responses after laser stimuli (De Tommaso et al., 2011). Cognitive deficiency includes damage to language, memory, attention and decision-making, which could result in abnormal behavior in HD patients (Singh and Agrawal, 2021). Slow pain progression might disturb sensory information integration and then lead to insufficient or inaccurate behavior in response to painful issues. An increasing number of studies have shown that HD patients experience pain but show a deficit in pain recognition. Because of motor and cognitive deficits, later-stage HD patients rarely complain of pain even with serious pain symptoms, suggesting the possibility of pain signal transfer delay or dysfunction (Albin and Young, 1988; Andrich et al., 2009; Baez et al., 2015). In addition to cognitive deficits, there are other behavioral abnormalities in HD, including emotional damage (psychosis, depression, anxiety) and empathy deficits, which cause negative pain recognition (Baez et al., 2015). The painful stimuli may interrupt sensorimotor information integration (Fil-Balkan et al., 2018; Millian-Morell et al., 2018) and lead to global deterioration of HD (De Tommaso et al., 2011; de Tommaso et al., 2016a).

There are still some other interesting findings concerning specific pain in HD. It has been reported that a series of abnormal pain events occur and change as the disease develops, including back pain, headache, limb pain, abdominal pain (Sprenger et al., 2021). In an elderly study, the relationship between gastroesophageal inflammation and HD was explored. Many HD patients have gastritis or esophagitis symptoms but without complaints, which has an obvious relationship with the duration and severity of HD (Andrich et al., 2009). The increase in abdominal pain might be highly related to the gastroesophageal disturbances in the end HD stage. Additionally, fractures seem to increase as HD progresses, which might be linked with bone density alteration (Carroll et al., 2015).

## Pain-related affecting factors

The correlation between pain and HD is interesting, involving a series of affecting factors. First, HD itself may cause pain and is rarely treated, partly due to the insufficient awareness of pain from physicians, which is supported by the incoherence between painful conditions and analgesic use (Sprenger et al., 2021). Second, neuronal loss or dysfunction could also cause a lack of self-awareness in HD, making it difficult to gather pain-related data through self-evaluation (Madariaga et al., 2021). Third, the incoherence between pain and HD might be clarified by basal ganglia disturbance, which could increase pain severity but with less or no pain behavior, probably owing to disorders in sensation, motor function and/or cognition (Borsook et al., 2010). Fourth, emotional factors are critical and closely linked with pain behavior in the HD population. A high frequency of emotional issues, such as depression and anxiety, are popular in the HD manifest periods (Sprenger et al., 2021). Finally, different pain criteria could cause different pain assessment data, which might underestimate the pain prevalence in the HD population. For example, specific dystonia is not considered a painful symptom, but it frequently appears and triggers pain in HD progression (Buhmann et al., 2020).

As HD progresses, an increasing number of affecting factors appear and are present, such as sex, illness stage, daily exercise, medicine and so on, and these factors interact with one another. Therefore, systematic studies are required to explore the detailed relationship between pain and HD in the future.

## Pain-related potential mechanisms

**FIGURE 2:**
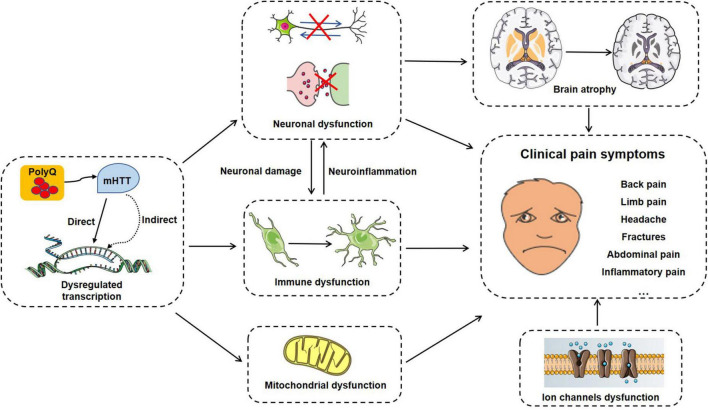
A summary of potential pain-related molecular mechanisms, cellular processes and clinical symptoms in HD progress.

As a typical genetic disease, HD is primarily characterized by motor and psychiatric dysfunction, and rarely focuses on pain abnormalities. Here, we outline some potential mechanisms associated with pain in HD, summarized in Figure 2.

### Brain degeneration and dysfunctions

The well-known characteristics of HD is progressive brain and basal ganglia atrophy, involving many functional regions and leading to motor and cognitive dysfunction as well as dementia and psychosis. Studies have reported that many regions of the brain are influenced in HD, including gray matter volume decrease, cortical thinning, and striatum deterioration.

Brain structures, such as the cortex, thalamus, cerebellum, and hippocampus, are essential areas related to pain sensory, function and functionally integrate with each other to modulate pain processing. However, the particular parts of brain atrophy associated with pain in HD are seldom considered. The brain regions involved in pain and HD are summarized and listed below (Table 1).

**TABLE 1:** The brain regions involved in pain and HD.

Brain regions	Connections with pain	Connections with HD	Clinical signs/meanings
Prefrontal cortex	Involved in acute and chronic pain, neuropathic pain, regional pain (Seminowicz and Moayedi, 2017; Ong et al., 2019; Huang et al., 2020)	Deterioration and dysfunction (Gomez-Anson et al., 2009; Gray et al., 2013; Matsui et al., 2014, 2015)	Signs: back pain, migraine, headache, trigeminal pain, complex regional pain syndromes Meanings: primary control center for descending pain modulation and pain relief (Seminowicz and Moayedi, 2017; Ong et al., 2019)
Anterior cingulate cortex	Involved in acute and chronic pain, neuropathic pain, inflammation pain (Bliss et al., 2016; Li et al., 2021; Wang et al., 2021)	Degeneration and neuron loss (Thu et al., 2010; Hobbs et al., 2011; Kim et al., 2014)	Signs: central sensitization, anxiety, neuropathic pain, visceral pain, inflammatory pain, transferred pain Meanings: contributes to multiple pains, mediates pain-related sensory and emotional responses (Li et al., 2021; Smith et al., 2021; Xiao et al., 2021)
Amygdala	Involved in acute and chronic pain, neuropathic pain, central pain, comorbid pain (Zhu et al., 2019; Hua et al., 2020; Neugebauer et al., 2020)	Reduced volume, poor motor and cognitive function (Mason et al., 2015; Lamirault et al., 2017; Ahveninen et al., 2018; Alexander et al., 2020)	Signs: visceral pain, muscle pain, neuropathic pain, arthritis pain, emotional-painful disorders: anxiety, depression, addiction, suicide Meanings: important for emotional pain dimension and modulation (Neugebauer, 2015; Thompson and Neugebauer, 2017; Neugebauer et al., 2020)
Hippocampus	Involved in chronic pain, cancer pain, inflammation pain, neuropathic pain (Li et al., 2020; Liu et al., 2021; Mai et al., 2021)	Reduced volume, synaptic and memory impairment (Harris et al., 2019; Wilkie et al., 2020; Glikmann-Johnston et al., 2021)	Signs: tactile allodynia, subacute and chronic back pain, abnormal mood, memory, cognition, stress responses, inflammation pain Meanings: mediates pain processing, pain-related attention, anxiety, stress (Vasic and Schmidt, 2017; Liu et al., 2018; Wei et al., 2021)
Gray matter	Involved in chronic pain, muscle pain, osteoarthritis pain (Malfliet et al., 2018; Barroso et al., 2020; You and Jackson, 2021)	Neuronal loss, atrophy and dysfunction (Faria et al., 2016; Johnson and Gregory, 2019; Sweidan et al., 2020; De Paepe et al., 2021)	Signs: central sensitization, pain sensitivity, pain cognitions disability, chronic spinal pain, osteoarthritis pain, musculoskeletal pain Meanings: mediates and reflects pain sensation, descends pain processing (Malfliet et al., 2018; Barroso et al., 2020; Ninneman et al., 2022)
Striatum	Involved in acute and chronic pain, inflammation pain, neuropathic pain (Barcelo et al., 2012; Jin et al., 2020)	Atrophy, neuronal loss and neurocognitive dysfunction (Sepers et al., 2018; Wilkes et al., 2019; Bulk et al., 2020; Lemoine et al., 2021)	Signs: Persistent pain, anxiety, sleep loss, anxiety, low back pain, pain-related fear Meanings: pain inhibition, takes part in the pain modulatory system (Martikainen et al., 2015; Krause et al., 2019; Boccella et al., 2020; Jin et al., 2020)

### Spinal cord and peripheral nervous systems dysfunctions

As major pain regulation areas, spinal cord (SC) and peripheral nervous systems (PNS) play vital roles in pain processing and transduction, also showed morphological and functional disorders in HD. SC gradual atrophy were detected and confirmed in manifest HD patients, as well as in the early stage (Muhlau et al., 2014; Wilhelms et al., 2017). One postmortem study detected the expression and location of mHTT in the SC of HD patients and found that mHTT preferred to express in the spinal gray matter (Sciacca and Cicchetti, 2017). The deterioration of gray matter and white matter in SC were also observed in HD mice. The expression of mHTT in oligodendrocytes resulted in myelination abnormalities, which can be rescued by mHTT reduction (Ferrari Bardile et al., 2021). The SC impairments in HD inevitably brought many sensory issues, such as pain. Abnormal pain signals in SC were observed along with a dysfunction of pain signal transportation, which finally induced sensory alternation in HD patients (De Tommaso et al., 2011; Perrotta et al., 2012). At SC level, mHTT could result in significant changes of pain behavior and pain-related cytokine in HD mice (Lin et al., 2018).

In SC and PNS, it was reported that glia cells participated in pain responses mainly through regulating pain signals transmission, neuroinflammation and neuron-glia interactions. Glial cells were highly involved in the development of HD processing. The special expression of mHTT in glial cells contributed to normal function lose and neuropathic pathology (Wilhelms et al., 2017; Wilton and Stevens, 2020). Both in HD patients and animal models, the cell morphology, metabolism and functions of astrocyte were greatly changed as well as their interactions with neurons (Skotte et al., 2018; Osipovitch et al., 2019). In addition, the abnormal expression of mHTT in astrocytes is greatly related with HD pathology motivation (Diaz-Castro et al., 2019; Wood et al., 2019). The cell size and structural changes of microglial were explored in HD, along with motility and migration disorders. Meanwhile, microglia cells in HD showed upregulated levels of inflammatory cytokines, contributed to pain processing and abnormity (Diaz-Castro et al., 2019; Liu et al., 2019). In HD mice, phenotypic and molecular studies revealed that the structural and functional deficits of oligodendrocyte appeared in HD early stage, causing severe oligodendrocytes’ dysfunctions, including thinner myelin sheaths, remyelination impairments and less response to demyelinating injury (Teo et al., 2016, 2019).

### Gene expression dysfunctions

One of the main pathological mechanisms in HD is transcription disorder. Many genes showed expression changes in HD (Lee et al., 2013; Sipione et al., 2016). Mutant Htt can interact with transcription factors, such as p53, CREB and PGC-1a, and disrupt cell survival, energy metabolism and protein expression (Jiang et al., 2006; Chaturvedi et al., 2012; Kim et al., 2016; Aravindan et al., 2020). As a key neurotrophic factor, the transcription, expression and transport of BDNF, which is essential for striatal neuron survival, are badly impaired in HD (Hong et al., 2016). Therefore, the stratum becomes the most vulnerable region due to a low level of BDNF (Park, 2018). In addition, synaptic dysfunction of the cortical- striatum is due to deficiency in the BDNF pathway via p75 (postsynaptic receptor), which is also thought to act with TrkB in HD (Pan et al., 2018).

Huntington-associated protein 1 (HAP1) is the earliest protein found to interact with Htt and contributes to cargo (vesicle, receptor, and neurotrophic factor) trafficking, the function of which is interfered with by mHtt (Jimenez-Sanchez et al., 2017), including the synthesis and release of BDNF in the cortex and the retrograde transport of TrkB in the striatum (Park, 2018). HAP1 is highly involved not only in HD but also in pain progression, and enriched in the spinal dorsal horn and dorsal root ganglia (Islam et al., 2017, 2020), which are considered the “primary sensory center”. In our study, we found that HAP1 could regulate Cav1.2 surface expression, which in turn influenced neuronal excitability, BDNF secretion, and inflammatory responses and ultimately modulated pain progression (Pan et al., 2023). HAP1 deficient mice exhibited mechanical allodynia and hyperalgesia inhibition in acute and chronic pain models (Gloor et al., 2022).

### Neuronal and synaptic dysfunctions

Neuronal abnormality is one of the initial pathological changes in HD, along with synaptic plasticity alteration, involving many factors, such as gene transcription, protein expression and transmission (Jimenez-Sanchez et al., 2017). As a scaffold protein, Htt contributes to vesicle transport through microtubules and motor proteins, while mHtt can inhibit the axonal delivery of cargoes and organelles, reduce neurotransmission and hinder signaling (Jimenez-Sanchez et al., 2017; Snowden, 2017; McColgan and Tabrizi, 2018). Axonal delivery is important for cargo transfer to membranes and expression. However, the transport of many aborted receptors, including GABA_A_ (γ-aminobutyric acid type A), AMPA (a-amino-3-hydroxy-5-methyl-4-isoxazolepropionic acid), and NMDA (N-methyl-d-aspartate) receptors, is found in HD, disrupting synaptic plasticity and excitability. Some studies have found that GABAergic neurons are reduced in neuropathic pain, and that elevated levels of GABAergic mediators and GABA_A_ receptors might reverse and attenuate pain (Knabl et al., 2008; St John Smith, 2018). In HD, redundant neurotransmission induced by NMDA receptors could cause neuronal death, specifically in striatal neurons. The function of NMDA receptors has been studied in multiple pain models, playing a pivotal role in attenuating central sensitization and pain hypersensitivity after stimulation (Kreutzwiser and Tawfic, 2019; Shin et al., 2020). Stimulated NMDA receptors can lead to calcium influx, which is critical for synaptic plasticity. AMPA receptors can regulate synaptic strength and plasticity and alleviate different types of pain hypersensitivity, including neuropathic (Wang et al., 2021), neuroinflammatory (Kopach et al., 2018), chronic (Bliss et al., 2016) and postoperative (Kopach et al., 2018; Khan et al., 2019; Kopach and Voitenko, 2021) pain. However, the balance between presynaptic, postsynaptic and extra-synaptic activities is altered in HD, termed excitotoxicity (Jimenez-Sanchez et al., 2017).

### Immune dysfunctions

Activated glial cells interact with neurons and affect pain processing by releasing neurotransmitters, molecules, inflammatory cytokines and chemokines. Immune dysfunction is an important factor in neurodegenerative disease. In HD, changes in immune cells and inflammatory responses have been discovered in the brain and peripheral system, indicating a potential role in pathogenesis (Andre et al., 2016). Increasing evidence has shown increased levels of proinflammatory mediators in early and late HD patients (Trager et al., 2014; Andre et al., 2016). When mHtt was chosen to be expressed in astrocytes, no notable changes appeared in glia or neurons, but mice showed pathological symptoms in the late stage. HD astrocytes present defects in the secretion of chemokines CCL5, BDNF and low levels of K^+^ channels, which can regulate neuronal excitability (Hong et al., 2016; Saba et al., 2020). In HD, nuclear factor-κB (NF-κB) acts as an inflammatory indicator and regulates the expression of various inflammatory cytokines, including IL-1 and TNF-α (Yusuf et al., 2021). NF-kB signals increase and evoke inflammatory responses in HD myeloid cells (Trager et al., 2014). In PNS, mutant huntingtin could influence inflammatory responses through NF-kB signaling inhibition. Downregulation of NF-κB attenuates inflammatory gene expression and suppresses inflammatory pain responses (Xiang et al., 2019; Yang et al., 2020; Kato et al., 2021). Microglial activation was discovered in the brains of HD patients and mice and is involved in neuronal degeneration. In addition, microglia expressing mHtt also exhibit decreased migration (Kwan et al., 2012; Crotti et al., 2014). Moreover, bone marrow transplantation could inhibit pathology by increasing the levels of cytokines and chemokines in HD mouse models (Jimenez-Sanchez et al., 2017).

### Mitochondrial dysfunctions

Mitochondria are involved in ATP production, calcium balance, and protein synthesis, which influence neuronal survival, growth and apoptosis (Dai et al., 2020; Doyle and Salvemini, 2021). Some studies have suggested that mitochondrial abnormalities are found in HD and are probably involved in the pain process (Jimenez-Sanchez et al., 2017). Some studies found that huntingtin mutation could cause mitochondrial calcium imbalance by binding with the outer membrane, interrupting the axonal transport of mitochondria and ultimately reducing ATP production (Shirendeb et al., 2011; Reddy and Shirendeb, 2012). Ca^2+^ is an essential signaling molecular, the abnormal activities of which play complex roles in HD (Jimenez-Sanchez et al., 2017), thereby leading to abnormal pain responses indirectly or directly. The fusion-fission cycles of mitochondria are regulated by dynamin-related protein 1 (Drp1), the deficiency of which can inhibit mHtt-evoked mitochondrial toxicity and delay HD development (Zhan et al., 2018; Dai et al., 2020). Drp1 and its associated mitochondrial malfunction have been reported to influence the pathological process of neuropathic pain (Jimenez-Sanchez et al., 2017; Zhan et al., 2018; Kun et al., 2019). Because of mitochondrial dysfunction, HD patients and models exhibit reactive oxygen species (ROS) (Jimenez-Sanchez et al., 2017), which have been observed and function in neuropathic pain (Flippo and Strack, 2017; Dai et al., 2020).

### Ion channels dysfunctions

Ion channels are considered as major targets for regulating pain sensation, due to their notable distribution in sensory neurons and key tissues. A lot of pain-related ion channels have been discovered and identified as potential pathogenic factors in HD, including transient receptor potential (TRP) channels, calcium (Ca^+^) channels, potassium (K^+^) channels, acid sensing ion channels (ASICs).

TRP channels are ubiquitously diffused in CNS, PNS and SC as well as dorsal root ganglia (DRG). TRP channels are mainly expressed in membranes of neurons and non-neuronal cells, mediating ion homeostasis and emerging as crucial pain transducers (Nilius and Owsianik, 2011; Moore et al., 2018; Lee et al., 2021). It had reported that TRP subfamilies TRPC (canonical) and TRPV (vanilloid) were associated with HD. Decreased level of endogenous TRPC1 was explored in HD striatal neurons, together with increased level of TRPC5 glutathionylation, leading to neurons apoptosis in HD mice (Hong et al., 2015, 2020). Furthermore, pharmacological experiments demonstrated the potential effects of TRPV1 channel in HD models, suggesting the possible contribution of TRPV1 in HD dysfunctions (Lastres-Becker et al., 2003).

Calcium channels have fundamental roles in neuronal health and functions and have a series of subtypes according different structures, pharmacological and physiological properties. L-type channels are abundantly expressed in CNS and SC, regulating Ca^2+^ influx and neuronal sensitization (Roca-Lapirot et al., 2018; Hopp, 2021). Its subtype Cav1.2 was highly involved in HD, whose expression and functions were interfered by mHTT (Miranda et al., 2019). The store-operated Ca^2+^ entry (SOCE), a major Ca^2+^ entry pathway, was greatly increased in HD and attributed to Ca^2+^ homeostasis disorder by activating mHTT indirectly (Czeredys, 2020). Besides, the interaction between mHTT and inositol-1,4,5-triphosphate receptors (IP3Rs) can lead to abnormally Ca^2+^ levels in R6/2 HD mouse, containing high intracellular Ca^2+^ level and low mitochondrial Ca^2+^ level (Mackay et al., 2018; Schrank et al., 2020). Furthermore, ryanodine receptor (RyR) associated Ca^2+^ abnormity had been investigated and documented in HD (Dridi et al., 2020).

Potassium (K^+^) channels are functional for pain processes by regulating pain transmission and modulation, whose dysfunction can induce a series of pain problems (Tsantoulas, 2015; Busserolles et al., 2016). In HD, K^+^ channels also have important roles in physiological and pathological conditions. Glia cells express many rectifying K + channel (Kir) subfamilies and function in HD processes (Zhang et al., 2018). In R6/2 and Q175 HD mouse models, Kir4.1 decrease can cause disturbances of astrocyte–mediated K^+^ homeostasis (Tong et al., 2014; Nwaobi et al., 2016). Evidence indicated that both HD patients and models showed obvious decrease of Kir4.1 level in astrocytes, leading to a high K^+^ extracellular concentration and neuronal damages (Nwaobi et al., 2016; Wang et al., 2022). Furthermore, K_ATP_ channels had been indicated to associate with abnormal neuronal firing in HD mice (Atherton et al., 2016; Rallapalle et al., 2021).

Additionally, acid sensing ion channels (ASICs) are highly expressed in nervous system, and greatly involved in both pain processing and HD development (Deval and Lingueglia, 2015; Sluka and Gregory, 2015; Zhou et al., 2016; Zhu et al., 2022).

## Conclusion and future perspectives

HD, as a classic neurodegenerative disorder, provides scientists with ideas for many interesting studies on social, financial and medical issues. Pain is one of the major features, a subjective and direct way to express discomfort and suffering. As HD progresses, pain can be produced directly by many elements, such as neuron loss, atrophy, muscle decline, and bone density reduction; however, subjective pain behavior might be limited or concealed owing to motor and sensory dysfunction and cognitive deficiency. Because of cognitive problems, HD patients suffer from pain but rarely complain, resulting in pain that is limited and neglected, therefore leading to underestimation and no treatments. As a result of insufficient attention, special evaluations and therapeutic guidelines are unavailable, and clinical treatments for pain are limited to analgesics and anti-inflammatory agents without a full speculation of potential mechanisms. However, in many other diseases, particularly AD and PD, specific scales and treatments are used and applied to pain issues, which are considered fully as a major symptom instead of a secondary problem.

This review notes that the major issues related to pain are medical neglect and carelessness, as attention and treatment are mostly focused on motor-related symptoms. Hence, health doctors and caregivers should pay more attention to pain problems during HD progression, especially in the later stage. The correlation between HD and pain should be further investigated. Current studies and evidence associated with pain in HD suggest a huge challenge and opportunity for scientists in the neuronal-pain field. Future studies should focus on pain-related factors and intrinsic mechanisms, pay more attention to specific scales, equipment, treatments and identify effective therapeutic methods for HD patients undergoing pain conditions.

## Author contributions

GC and JP designed and drafted the manuscript. All authors contributed to the manuscript revision and approved the final version.
